# State-of-the-Art Review on Removal of Naturally Occurring Radioactive Materials in Water

**DOI:** 10.3390/ijerph22050727

**Published:** 2025-05-02

**Authors:** Zaid Al-Shomali, Alcides Pereira, Ana Clara Marques, Maria de Lurdes Dinis

**Affiliations:** 1 CERENA-FEUP—Centre for Natural Resources and the Environment, Engineering Faculty, University of Porto (FEUP), Rua Dr. Frias, 4200-465 Porto, Portugal; mldinis@fe.up.pt; 2 Laboratório de Radioatividade Natural, Faculdade de Ciências e Tecnologia da Universidade de Coimbra (LRN-FCTUC), Rua Sílvio Lima, Polo II, 330-790 Coimbra, Portugal; apereira@ci.uc.pt; 3 CERENA, DEQ, Instituto Superior Técnico, Universidade de Lisboa, Avenida Rovisco Pais, 1049-001 Lisboa, Portugal; ana.marques@tecnico.ulisboa.pt

**Keywords:** naturally occurring radioactive materials, water treatment, radionuclide removal techniques

## Abstract

Radionuclide-contaminated water is carcinogenic and poses numerous severe health risks and environmental dangers. Thus, effective removal techniques are required to ensure the safety of drinking water sources. This article overviews several methods to remove naturally occurring radioactive materials (NORMs) from water, including adsorption, coagulation, reverse osmosis, ion exchange, electrodialysis, iron manganese filtration, and membrane filtration. A search is conducted in different scientific databases to identify relevant articles, reviews, and studies on removing radionuclides from water. The overarching goal of this article is to deepen the understanding of the techniques available for radionuclide removal from water and to foster the creation of innovative solutions for water contamination concerns. Each technique is examined in terms of its efficiency, cost-effectiveness, and sustainability in removing specific radionuclides from water sources. The advantages and limitations of these techniques are discussed, highlighting the importance of selecting the most appropriate method based on the characteristics of the radionuclides and the water source. Different methods can be combined for the more effective removal of radionuclides from water, such as coagulation and filtration, reverse osmosis, and ion exchange. The treatment of water contaminated with radionuclides requires prior laboratory work and pilot-scale tests to determine the most suitable, cost-effective, and environmentally friendly method.

## 1. Introduction

Natural radionuclides in drinking water pose significant health hazards to humans and ecosystems. Radionuclides, such as uranium and its progenies and thorium and its progenies, can be present in water sources due to natural geological processes, industrial activities, and accidental release. Consequently, effective removal and water treatment methods are crucial to ensure the safety and quality of water bodies, particularly drinking water, as radionuclide content can result in both short-term and long-term health risks.

Different naturally occurring radionuclides can be found in many groundwater sources, thus increasing the risk of developing various types of cancer. For instance, radon, the progeny of uranium decay, when inhaled over prolonged periods, can cause lung cancer [1]. To date, epidemiological studies have not confirmed an association between the ingestion of drinking water containing radon and an increased risk of stomach cancer. When radon is dissolved in drinking water sources and released into the atmosphere, it increases health risks. The World Health Organisation and epidemiological studies performed by the US-EPA have confirmed the health risks posed by radon inhalation.

Exposure occurs from the inhalation of radon rather than from ingestion [2]. Conventional water treatment techniques, like coagulation, sedimentation, and filtration, may need additional treatment stages to remove radionuclides, like nanofiltration, reverse osmosis, and electrosialysis. Therefore, advanced treatment technologies have been developed, with some still under development, to manage the risk of hazards that may compromise the safety of drinking water, which concerns public health. These methodologies comprise adsorption, ion exchange, membrane filtration (e.g., ultrafiltration, nanofiltration, and reverse osmosis), and precipitation/co-precipitation processes. Each method has advantages and limitations regarding removal efficiency, cost-effectiveness, and practical application.

This state-of-the-art review aims to critically assess the current knowledge on radionuclide removal from water and highlight several treatment methods’ strengths and limitations. By synthesizing existing research, this review aims to provide insights and directions for further advancements in the field, ultimately contributing to developing more efficient and sustainable radionuclide removal technologies for water purification.

## 2. Background

### 2.1. Origin and Sources of Natural Radioactivity

Natural radiation originates from terrestrial, cosmic, and internal radiation (the human body). Radioactivity is the property exhibited by certain types of matter that spontaneously emit energy and subatomic particles.

Internal radiation sources involve radioisotopes, such as potassium-40, lead-210, and carbon-14, which are present in the environment [3]. Additionally, cosmic radiation from the sun and stars constantly impacts the Earth. Cosmic radiation varies according to the Earth’s magnetic field and the elevation of different regions of the world [3]. Approximately 82% of the radiation dose comes from natural sources, as follows: 55% from radon, 8% from cosmic radiation (from the sun and stars), another 8% from terrestrial sources (radioactive material in rocks and soil), and 11% from internal sources (radioactive materials, primarily potassium-40, from food and water consumed in the daily diet). The remaining 18% of the dose comes from anthropogenic (man-made) sources such as medical X-ray exposure (11%), exposure from nuclear medicine procedures (4%), consumer products (3%), and other sources (<1%). These other sources include occupational exposure, nuclear fallout, and the nuclear fuel cycle [3].

Natural radiation varies worldwide, with regions containing high-volume uranium and thorium in their soils potentially presenting higher radiation dose levels.

There are two forms of radiation, as follows: ionizing and non-ionizing. Ionizing radiation has enough energy to remove electrons from atoms and molecules (ionize), while non-ionizing radiation does not have enough energy to ionize atoms and molecules. The four major types of ionizing radiation encompass alpha (α), beta (β), gamma (γ), and neutron radiation. Alpha decay leads to the loss of two neutrons and two protons [4]; beta decay emits electrons (β− decay) or positrons (β+ decay), while γ decay releases electromagnetic radiation from the nucleus. Alpha particles are the heaviest form of ionizing radiation and have the least penetrating capability. Beta particles can penetrate deeper into materials than α particles, while γ rays can pass through most materials, including concrete and lead, unless these present a certain thickness [4]. Neutron radiation involves the ejection of neutrons from unstable nuclei through nuclear fission (a reaction that splits the nucleus into smaller elements) and other processes [5]. Radionuclides in the Earth’s crust, such as uranium, thorium, and their progenies, undergo radioactive decay, emitting alpha, gamma, and beta radiation. The three natural radioactive decay series are ^232^Th, ^238^U, and ^235^U chains. ^238^U forms 99.3% of natural uranium, ^235^U constitutes 0.6945, and the abundance of ^234^U is 0.0055% [6].

The half-life of a nuclide is the time required for half of the atoms of a particular radioisotope to decay into another isotope. A specific half-life is a characteristic property of each radioisotope. The half-lives of ^235^U and ^238^U are 710 million years and 4.5 billion years, respectively [6]. Key progenies of ^238^U include ^226^Ra (half-life of 1600 years) and ^222^Rn (half-life of 3.823 days) [6]. The half-life of ^232^Th is around 14 billion years, and its principal progenies comprise ^224^Ra and ^222^Rn. The half-lives of ^224^Ra and ^222^Rn are 3.62 days and 55.62 s, respectively [6].

Decay involves the disintegration of radionuclides and sequentially producing progenies until a stable nucleus remains. In the decay process, the resulting isotope in each series determines whether disintegration occurs via beta or alpha emission and reflects the half-life (Table 1, Table 2 and Table 3 indicate the decay series and half-lives of ^235^U, ^232^Th, and ^238^U decay chains).^238^U emits the ^222^Rn isotope in its decay chain, producing lead as a stable isotope at the end of the chain [7]. Table 1, Table 2 and Table 3 show the decay series of uranium-238, thorium-232, and uranium-235, respectively.

### 2.2. Physicochemical Characteristics of Radon, Radium, Thorium, and Uranium

To encourage creative solutions for water polluted with radionuclides, their physicochemical characteristics should be studied, as these characteristics relate to the reactivity, solubility, and mobility of the radionuclides under investigation. Table 1 summarizes these characteristics.

### 2.3. Decay Series of Uranium-238, Thorium-232, and Uranium-235

Table 2, Table 3 and Table 4 indicate the decay series and half-lives of ^235^U, ^232^Th, and ^238^U decay chains.

**Figure 1:**
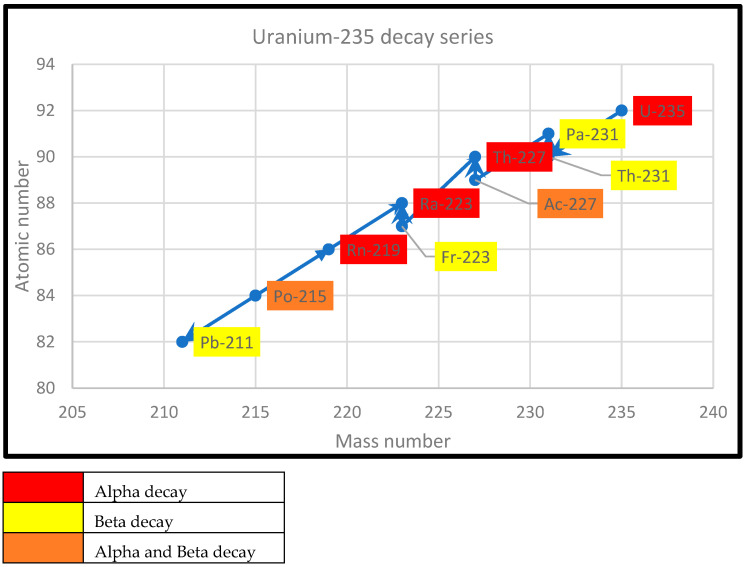
Uranium-235 decay series.

**Figure 2:**
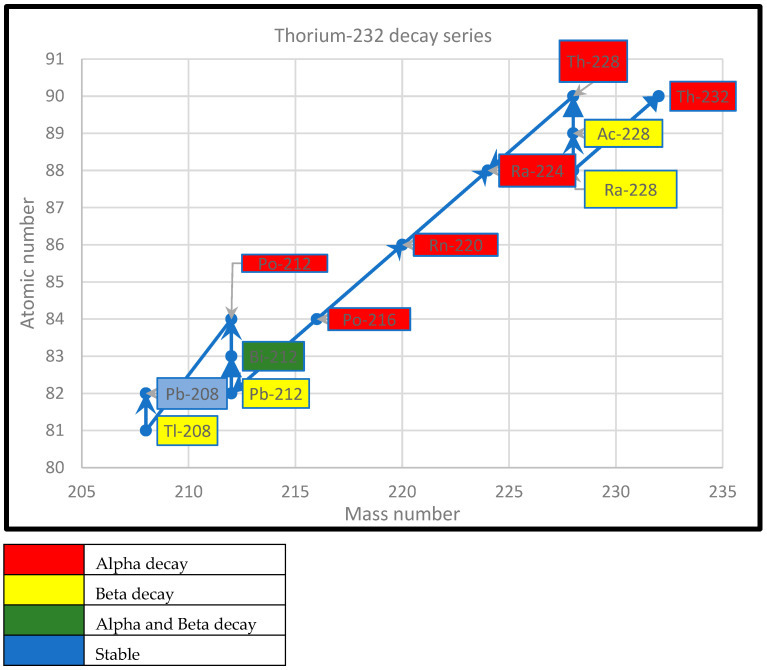
Thorium-232 decay series.

**Figure 3:**
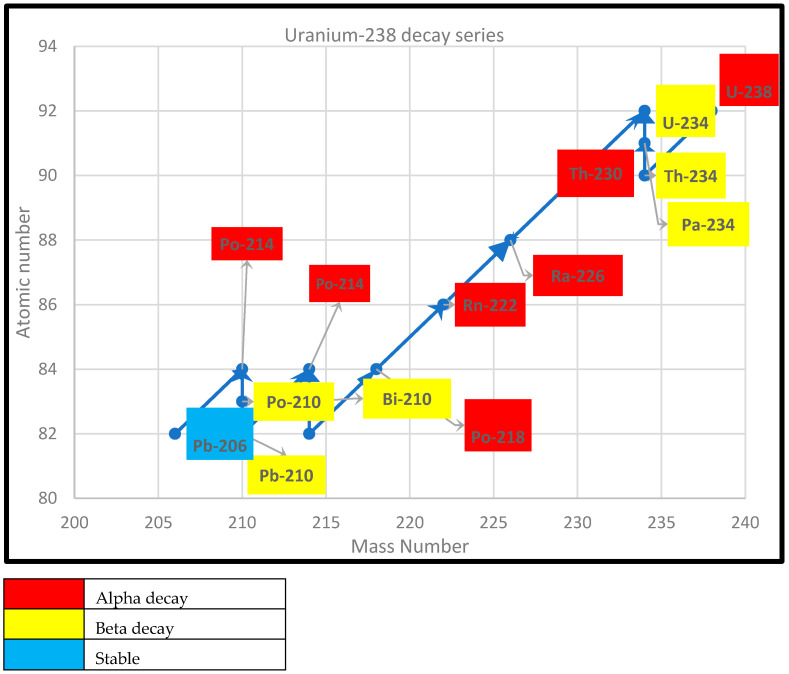
Uranium-238 decay series.

Graphical representations of the decay of ^235^U, ^232^Th, and ^238^U are presented in Figure 1, Figure 2 and Figure 3.

In light of uranium-238, uranium-234’s half-life is 245,500 years, while it takes 164 microseconds for the mass of Po-214 to reduce by half. Products of the natural decay chain of uranium-238 account for over 50% of the global annual dose [19]. ^222^Rn, a radiogenic radionuclide of the ^238^U decay series, comprises 55% of the world’s radiation dose [19].

Soils and rocks contain low levels of uranium, thorium, and their decay isotopes (the thorium and uranium concentrations in rocks are less than 0.1%) [19]. The upper continental crust content of uranium is 2.7 ppm, while that of thorium is 10.5 ppm. Potassium-40, a radioactive isotope, occurs in trace amounts in potassium minerals [19]. The average crustal abundances of uranium and thorium are approximately 2.5 and 10 ppm, respectively. A third important radioelement is the potassium isotope, ^40^K [20]. All of these are found in varying concentrations in most rock-forming minerals. Although ^40^K represents only 0.0118% of natural potassium, it contributes significant radiation because of the relatively high abundance of potassium in rocks (approximately 2.5% of the Earth’s crust [20]). The average abundances of thorium-232, uranium-238, and potassium-40 in soil are 9 ppm, 1.8 ppm, and 1.5 ppm, respectively [20]. Radium concentrations vary according to rock types. According to the worldwide average, the volume of radium in the lithosphere is around 33 Bq/kg [21]. Igneous rocks have a maximum of three times higher activity concentrations of radium-226 than limestone and sandstone [21]. The worldwide abundance of ^222^Rn in soil ranges between 4 and 40 kBq/m^3^ [20,21].

### 2.4. Natural Radionuclides in Water

Radium-226, a decay isotope of thorium-230, is a radionuclide of concern in different water sources. Usually, groundwater contains significantly higher radium-226 concentrations than surface water [21]. Oceans contain lower amounts of radium-226 than groundwater and surface water. The radium-226 activity concentration in oceans ranges from 4 to 19 Bq/m^3^ [22]. Some studies have shown that the average radium concentration in groundwater can range from 50 Bq/m^3^ to 900 Bq/m^3,^ depending on the geology. Radium-226 decays to radon-222, which may be dissolved in water bodies. The ^222^Rn content in groundwater may present a broad spectrum of activity concentrations ranging from 1 to 1000 kBq/m^3^ [21]. The ^222^Rn concentration in soil depends on the radium content in rocks, its emanation rate, humidity, and porosity. The ^222^Rn concentration in groundwater varies significantly across the world. There are considerable differences in the ^222^Rn concentration levels in groundwater in Portugal and Europe. In 2016, a study was conducted in Portugal on the exposure to indoor ^222^Rn levels in Portuguese thermal spas [23]. The study, which spanned 16 thermal spas, involved the measurement of radon-222 in air and thermal mineral water twice yearly from 2012 to 2016.

In the tested water boreholes at the thermal spas, the radon-222 concentration in the water samples extracted ranged between 41 Bq/L and 6949 Bq/L [24]; it was clear that the water samples collected showed values of over 100 Bq/L, which is the recommended reference value of the Council Directive 2013/51/EURATOM. The extended study showed that the indoor ^222^Rn concentration varied between December 2018 and November 2019 from 202 to 1941 Bq/m^3^ in one thermal spas and 937 to 1750 Bq/m^3^ in another thermal spa [24]. Approximately 60% of the values obtained for the radon-222 concentration in indoor air exceeded the reference level of 300 Bq/m^3^ [24].

In La Palma, Spain, the concentration of radon-222 in groundwater wells ranged between 500 and 5810 Bq/m^3^ [25]. In addition, other areas presented radon concentrations varying from 71 to 20,980 Bq/m^3^ [26]. In Poland, the radon-222 concentrations in air in prone regions (geographic areas or administrative regions defined based on surveys indicating a significantly higher radon-222 concentration than in other parts of the country) [27] ranged from 35,300 to 272,000 Bq/m^3^ [27]. The activity concentrations for radon-222 in Finland for raw groundwater varied from 50,000 to 100,000 Bq/m^3^ [28].

### 2.5. Radionuclides in Drinking Water Supplies

Natural radionuclides worldwide include radium, radon, and uranium isotopes in many drinking water supplies. Different water quality guidelines are applied outside of European countries. Table 5 summarizes the water quality guidelines used in Africa, Asia and the Pacific, Europe, Latin America, North America, and West Asia to control ionizing radiation in drinking water.

US regulations require community water systems to maintain radionuclides below the maximum contaminant levels (MCLs). The combination of radium-228 and radium-226 should have an MCL of 185 Bq/m^3^. Uranium content should not exceed 0.372 Bq/m^3^ [29]. Other natural radionuclides are controlled under gross alpha and beta radioactivity. Gross alpha and gross beta refer to the radioactivity emitted by alpha and beta particles, respectively. The Euratom Drinking Water Directive (2013/51/Euratom) sets parametric values for some radioactive substances [30].

Parametric values should not be regarded as limit values. If monitoring water intended for human consumption indicates non-compliance with a parametric value, whether that poses a risk to human health should be considered. In this case, it may be necessary to take remedial action to improve the quality of the water to a level which complies with the requirements for protecting human health from a radiation protection point of view. Parametric values are applied in the following three categories: radon and tritium concentrations and indicative doses in drinking water [30]. “Indicative dose” refers to a reference level for annual radiation exposure, specifically from drinking water [31]. The parametric concept applies to tritium (100,000 Bq/m^3^), radon (100,000 Bq/m^3^), and an indicative dose of 0.1 mSv, as shown in Table 5.

The Jordan Institute of Standards and Metrology (JISM) collaborated with the Ministry of Health (MOH) to review and develop drinking water standards, including for the control of radioactive substances. JISM and MOH provide standard values rather than setting specific maximum limits for radionuclides [32]. The relevant agencies must investigate the source and type of radionuclides if beta and alpha radionuclides exceed the standard values. The European guidelines also indicate the derived concentration for ^210^Pb, although this substance is not explicitly regulated in the United States.

### 2.6. Regulatory Framework for Drinking Water Sources

The percentage of surface and groundwater that serves as drinking water sources varies worldwide. The total water abstracted each year in Portugal for groundwater and surface water sources is 540,785 hm^3^, with drinking water comprising 14.56% of this volume [33]. In Portugal, groundwater accounts for 60% of drinking water supplied [33]. Nevertheless, the country demonstrates an unequal groundwater supply due to precipitation and geological factors. The percentages of surface and groundwater sources rated as having a poor chemical status are 35% and 20%, respectively [33,34].

The primary source of drinking water in Jordan is groundwater. The drinking water sources in Jordan include groundwater (53%), surface water (32%), and treated wastewater (15%) [35]. Groundwater in Jordan poses health risks due to the presence of natural radionuclides. Radium-228 and radium-226 are the predominant radioactive isotopes in the Disi aquifer in Jordan [35]. The average radium-226 and radium-228 concentrations in the Disi aquifer are 0.516 and 0.287 Bq/L, respectively [36]. The radium-228 concentration is from two to three times higher than the World Health Organization’s (WHO’s) minimum recommended limit (MRL). The activity ratio (radium-228/radium-226 = 0.55) is five times higher than the WHO’s MRL [35,36]. Therefore, the Disi water requires treatment before being supplied to the public for drinking.

### 2.7. Regulatory Framework

The European Union (EU) and global policies have implemented a framework for radionuclides in water within the EU member states. In 1957, many EU countries, including the Netherlands, Italy, France, and Germany, signed the Euratom Treaty, which required parties to reduce environmental radiation emissions. Article 35 mandated states to implement essential strategies to monitor the radioactivity levels in water, soil, and air, complying with fundamental safety standards [37]. The most significant regulation establishing radionuclide control in water is Council Directive 2013/51/Euratom. Member states should apply remedial actions if radionuclide concentrations exceed parametric values. Data for these parametric values are presented in Table 5. The parametric value for tritium and radon-222 is 100,000 Bq/m^3^ (each considered separately). The maximum limit for ^222^Rn set by the EU can exceed 100,000 Bq/m^3^, but it should be lower than 1,000,000 Bq/m^3^ [30,37]. Other radionuclides are included in the indicative dose category. Council Directive 2013/51/Euratom provides the derived concentrations of radioactive isotopes in the indicative dose category for EU member states to ensure that the effective dose rate for public exposure does not exceed the total annual dose of 1 mSv [30,37]. The derived concentrations for some radionuclides include uranium-238 (3000 Bq/m^3^), radium-226 (500 Bq/m^3^), and radium-228 (200 Bq/m^3^) [30,37].

The International Atomic Energy Agency (IAEA) provides Basic Safety Standards (BSS 2014), containing guidelines for the member states to protect individuals from the harmful impacts of ionizing radiation. The guidelines for controlling radionuclides in drinking water require that the acceptable annual limit for public exposure to ionizing radiation should not exceed 1 mSv [38,39].

## 3. Materials and Methods

A thorough search was conducted in scientific databases such as PubMed, Scopus, Web of Science, and Google Scholar to identify relevant articles, reviews, and studies on removing radionuclides from water. The search keywords included “radionuclides removal”, “water treatment”, “radioactive contaminants removal”, and “removal of radionuclides from water” from 2010 to 2024. The search resulted in forty-five scientific articles, four conference papers, two reports from the European Commission, three reports from the United Nations Economic Commission, three reports from the International Atomic Energy Agency, one report from the US Environmental Protection Agency, one report from the US Nuclear Regulatory Commission, two reports from the United Nations Scientific Committee, and two reports from the National Council on Radiation Protection and Measurements. Articles for this review were chosen based on rigorous criteria, focusing on methods to remove radionuclides from water, and only studies related to water treatment applications were considered. Information extracted from the selected studies included the specific radionuclides targeted, the method used for their removal, its effectiveness in radionuclide removal efficiency, and any reported challenges or limitations. Data on experimental setups, conditions, and results were also gathered. The extracted data on different methods for radionuclide removal were analyzed for effectiveness, cost, applicability to different radionuclides, ease of implementation, and environmental impacts. A comparative analysis was conducted to evaluate the advantages and limitations of each removal method. The key findings from the selected studies were synthesized to present a comprehensive overview of radionuclide removal methods from water. The implications of these findings for water treatment practices and potential future research directions were discussed based on the synthesized literature.

The collected data, analysis, and synthesized findings were compiled and are presented in the following sections.

## 4. Techniques for Treating Radionuclides in Water

There are several processes and techniques for treating radionuclides in water, considering whether these radionuclides are soluble or insoluble in water.

These techniques can be divided into the following two main groups: physical methods for removing solids that are insoluble from uncontaminated water and chemical techniques for treating water that is contaminated with soluble forms of radionuclides [38]. Among the physical processes are microfiltration, ultrafiltration, nanofiltration, reverse osmosis, and evaporation. These methodologies involve water movement through a membrane or gas–liquid contact to retain dissolved contaminants in concentrated brine. Since energy efficiency is a significant consideration for these techniques, removing dissolved species is preferred over indiscriminate concentration.

Chemical methods, on the other hand, are typically selective in molecular separations. These methods encompass chelation, ion exchange, precipitation, electro-deionization (EDI), and solvent extraction. They target ions based on their chemical reactivity, solubility, partition coefficient, affinity for functionalized or charged surfaces, or response to electric fields. Among these, EDI is the sole chemical method involving electrochemistry and can function continuously without additional solvents. The study carried out by Alkhadra et al. (2022) [40] outlined the limitations of traditional chemical methods, indicating that while they have proven well-established and cost-effective in desalinating ocean water and concentrated solutions, their efficiency and energy consumption become problematic when applied to processes that dilute feeds or selectively eliminate specific contaminants from concentrated feeds [40]. In such cases, it is more advantageous to selectively remove trace amounts of the desired species rather than accumulating all dissolved species in a concentrated solution. Such techniques include various innovative methods based on electrosorption or electrokinetics for ion separation and water purification, consequently giving rise to advanced electrochemical methods [41].

Table 6 summarizes the primary techniques for removing radium, radon, and uranium from water.

### 4.1. Membrane Filtration Technology

**Figure 4:**
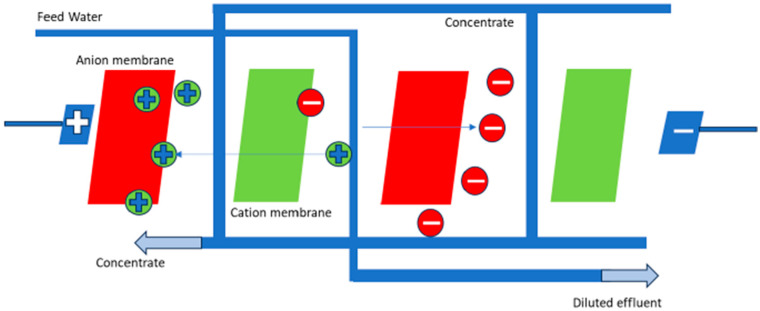
Electrodialysis reversal process.

Membrane-based treatment methods have become increasingly important for the removal of radionuclides from drinking water. Ultrafiltration, reverse osmosis, and electrodialysis reversal have emerged as the most widely utilized and effective approaches. Removing radionuclides using membrane treatment involves the application of semipermeable membranes that selectively filter out contaminants, including radionuclides, based on their size, charge, and molecular properties [42]. These membranes act as physical barriers, allowing purified water molecules to pass through while retaining radionuclides and other undesirable substances. Other studies have investigated the efficacy of membrane-based treatment technologies, such as reverse osmosis (RO), ultrafiltration (UF), and electrodialysis reversal (EDR) (Figure 4), for the removal of radionuclides from water sources [42]. A pilot-scale facility was employed to evaluate the performance of these membrane-based methods [42].

Four different scenarios were tested, and samples were collected at various stages of the treatment process. The collected samples were then analyzed to determine the concentrations of uranium, gross beta, and gross alpha. The findings demonstrated that RO and EDR significantly improved the topic of discussion about the assessment of radiological water quality, specifically focusing on the evaluation of removal rates surpassing 60%. Nevertheless, ultrafiltration did not demonstrate a substantial removal efficiency for uranium radioactivity, gross beta, and gross alpha. Based on the results obtained from the pilot-scale facility, an industrial-scale treatment facility, known as the SJD WTP, the Abrera and Sant Joan Despí water treatment plant, which withdraws water from the River Llobregat in the metropolitan area of Barcelona, was designed and constructed. Due to its superior performance in reducing radiological parameters, RO treatment was selected for implementation at the SJD WTP. Reverse osmosis treatment at the SJD WTP resulted in remarkable decreases in gross beta and gross alpha activity concentrations. On average, the RO treatment achieved removal rates of approximately 93% for gross beta activity and 95% for gross alpha activity at the treatment facility.

Table 7 explains the five main steps of a typical reverse osmosis system.

**Figure 5:**
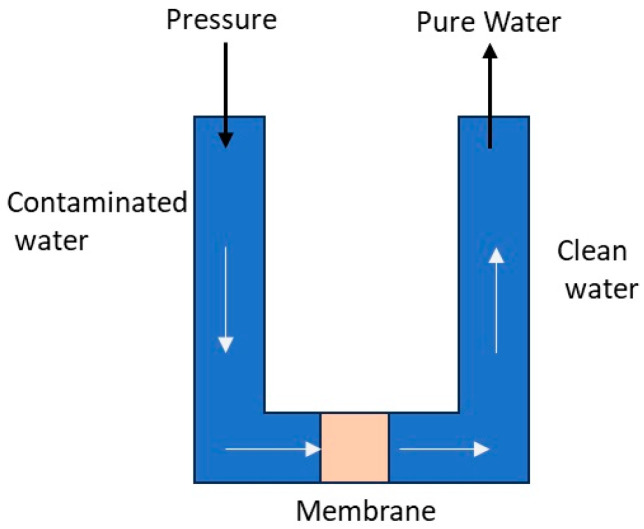
Reverse osmosis process.

While reverse osmosis (Figure 5) exhibits a high removal efficiency for a wide range of contaminants, it has several drawbacks, such as high capital expenditures, mature infrastructure requirements, and difficulty in downscaling due to the need for high-pressure pumps and robust plumbing, as mentioned by Alkhadra et al. [41]. Addressing the limitations of treating brackish water and dilute feeds using conventional membrane-based technologies requires considering reduced energy requirements and adaptive infrastructure as a viable approach [40,41,42]. Other studies assert that these membrane technologies have demonstrated their feasibility and high efficiency in removing a wide range of contaminants, including perfluoroalkyl substances (PFASs) [43], which are known for their persistence and resistance to traditional water treatment methods. However, the current RO and NF membranes must consistently meet the established guidance limits for PFASs in drinking water. They often face fouling issues that hinder their large-scale application. The authors intimate that novel strategies are being explored to overcome these challenges and comply with the increasingly stringent regulations concerning PFASs in drinking water. One such approach involves using nanocomposite mixed-matrix membranes (MMMs) that utilize advanced materials to revolutionize RO/NF technologies for water purification. Specifically, incorporating metal–organic frameworks as adsorbent fillers within polymeric membrane matrices presents a viable approach for selectively capturing and removing PFASs from water. The use of membrane technology, particularly high-pressure nanofiltration (NF) membrane systems and reverse osmosis (RO), to remove radionuclides from water is relatively limited and not extensively discussed in the literature. This study aims to update the literature on several techniques used in this field [43]. However, it is generally acknowledged that RO/NF membranes can achieve a removal efficiency of at least 95% for radionuclides while improving other relevant water quality parameters. The salt concentration in brine significantly increases during this process, but the overall removal efficiency for salts exceeds 99%. However, it is noted that a drawback of membrane technology is the generation of highly concentrated radioactive wastewater, known as retentate. Retentate is sometimes called the concentrate or the reject from the membrane process, i.e., the portion of the feed solution that is retained or not passed through the membrane. Retentate constitutes approximately 15–25% of the total flow and needs to be disposed of as low-level radioactive waste [43]. Additional variables that should be considered in reverse osmosis (RO)-based systems for radium removal include the necessity of pretreatment and the associated financial implications in terms of both capital and operating expenses. Nonetheless, ongoing advancements in membrane technology are making RO systems more competitive for radionuclide removal applications [43].

### 4.2. Nanotechnology

Despite their widespread use, traditional water purification methods are ineffective in removing radionuclides and require improvement in achieving adequate decontamination. Additionally, these conventional approaches are known to be costly and environmentally unsustainable. In light of these challenges, nanotechnology is an emerging method for addressing these concerns. Characterized by their unique properties, nanomaterials demonstrate enhanced capabilities for treating contaminated water, primarily credited to their faster absorption rates due to their large surface areas [42,43,44]. Current research is focused on the current state of nanomaterials, looking deeply at their efficacy as next-generation water purification systems. Furthermore, particular pressure is placed on their capability to remove trace elements and radioactive contaminants from aqueous solutions; this provides an overview of nanomaterials’ current state and prospects for eliminating trace elements and radionuclides from water [42,43,44].

However, nanostructures have appeared as efficient sorbents for water decontamination. Metal oxides, such as cerium oxide, titanium dioxide, and iron oxide, exhibit excellent adsorption capacities for heavy metals and radionuclides. Metallic–organic frameworks (MOFs) offer a wide range of tunable structures and pore sizes, enabling the efficient removal of contaminants. Nanoparticle-impregnated membranes, such as graphene oxide and carbon nanotubes, demonstrate exceptional water purification capabilities due to their significant specific surface area and exceptional mechanical strength. The unique properties of nanomaterials enhance their high efficiency in removing heavy metals and radionuclides from water. Their large surface area allows for increased contact with contaminants, promoting better adsorption and removal. Moreover, the high tensile strength of nanomaterials ensures their durability and long-term effectiveness in water treatment applications.

**Figure 6:**
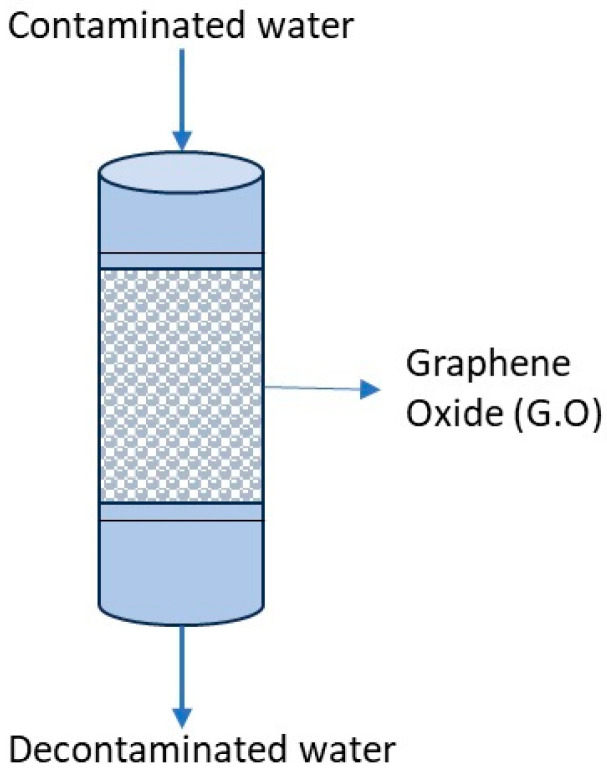
Graphene oxide water treatment diagram.

Tee et al. (2022) [45] provided a broad overview of nanomaterials for eliminating trace elements and radionuclides from water [45,46]. However, the author did not investigate specific nanomaterials and their synthesis methods or conduct an in-depth comparative performance analysis. The review focused on nanomaterials’ general capabilities and characteristics, without providing specific quantitative data on their efficiency in removing heavy metals and radionuclides. Detailed studies and experiments are needed to quantify their adsorption capacities, selectivity, and long-term stability. To remedy this lack of specificity, the research developed by Laver (2020) [47] focused on using graphene oxide (GO) as a sorption material for removing radionuclides and trace elements from contaminated water [47]. GO and reverse osmosis are compared in Table 8. A laboratory study of GO yielded positive results, demonstrating that it can be an effective and cost-efficient method for water purification. Graphene oxide can be produced using readily available equipment and easily accessible chemicals, making it a scalable and environmentally friendly option [47]. Graphene oxide is a two-dimensional carbon material that can be exfoliated into thin sheets in water. It possesses hydrophilic properties due to its oxygen-containing functional groups, allowing it to disperse quickly in water [47]. Graphene oxide’s vast surface area and reactive oxygen groups make it suitable for extracting heavy metals from water (Figure 6).

Laver (2000) [47] explored a GO molecular model developed using Density Functional Theory (DFT), which aligns with experimental data and existing theoretical studies. The model indicated that the stability of functional groupings, including oxygen on graphene surfaces, remains consistent regardless of the size of the nano-flake. Additionally, the DFT calculations suggested that epoxide group production at modest levels of surface oxidation is improbable, which aligns with experimental findings [48].

Furthermore, GO is more efficient in various conditions than other sorbent materials and extraction methods. For example, Sitko et al. (2013) [49] reported that the graphene oxide capacity for Cu (II) is significantly higher than other available methods [49]. Graphene oxide has shown a maximum sorption capacity of 294 mg g^−1^ for Cu (II) elimination, while conventional sorbent materials like activated carbon and carbon nanotubes exhibit much lower capacities [49].

Other materials, such as zeolite and algae, have been explored as alternative sorbents, but exhibit lower capacities compared to GO [48,49]. Similarly, zinc oxide nanoparticles (ZnONPs) have garnered focus as effective adsorbents for water decontamination due to their biocompatibility, affordability, stability, and antibacterial properties. Several research studies have been conducted on the adsorption of trace elements and radionuclides onto zinc oxide nanoparticles. Still, more comprehensive information must be given regarding the adsorption process’s kinetics, thermodynamics, and isotherms.

Akpomie et al. (2022) [50] addressed this gap by providing valuable insights into the adsorption capacity, reusability, and system for the adsorption of trace elements and radionuclides onto zinc oxide nanoparticles [50]. One characteristic that makes ZnONPs ideal in water treatment is their high adsorption capacity. Akpomie et al. (2022) [50] found that the adsorption capacity of ZnONPs is an attribute of the adsorbent and can vary depending on the experimental conditions and the type of pollutant [50]. Different synthesis methods and experimental parameters influence adsorption capacity. ZnONPs have high adsorption capacities for contaminants such as thorium, lead, copper, cadmium, nickel, and barium [50]. The increased adsorption capacity of ZnONPs for Cu (II) indicates a higher level of selective adsorption [50]. This selectivity can be attributed to the solid binding strength between ZnONPs and heavy metal ions [50]. This is influenced by the metal ions’ electronegativity and the surface hydroxyl groups on zinc oxide nanoparticles [50]. The adsorption mechanism of heavy metals onto ZnONPs involves various interactions, such as electrostatic, ion exchange, precipitation, chelation, reduction, hydrophobic, complexation, hydrogen bonding, and Van der Waals interactions [50]. The specific mechanism depends on the nature of the adsorbate and the surface features of ZnONPs. Furthermore, the reusability of ZnONPs is crucial for their practical application. Desorption and reusability studies have indicated the nature of the adsorbent–adsorbate interaction and the possibility of regeneration. ZnONPs have been successfully regenerated and reused for multiple adsorption–desorption cycles, especially for metals like lead, cadmium, cobalt, palladium, barium, and selenium. However, Akpomie et al. (2022) [50] noted that poor regeneration and reuse have been observed for copper and chromium, highlighting the need for alternative desorbing agents [50]. ZnONPs demonstrate significant adsorption capacities in water treatment for trace elements and radionuclides [50]. Further research is needed to explore their applicability for pollutants like manganese and radionuclides, such as radon, radium, and other naturally occurring radioactive materials. Additionally, more studies on reusing ZnONPs and regeneration are required to optimise their practical application.

### 4.3. Electrochemical Methods

In recent years, electrosorption and electrokinetics methods have emerged as viable electrochemical methods for aqueous purification and ion separations. These methodologies have paved the way for various emerging electrochemical methods. As discussed earlier Alkhadra et al. (2022) [41] comprehensively reviewed electrochemical methods [41]. A notable advancement is the discovery of deionisation shock waves in microchannels and porous media, which has sparked research on electrokinetic methods for deionization. Concurrently, the authors asserted that materials science has revealed new electrode chemistries that promote the electrosorption of ions through Faradaic effects, replacing conventional carbon-based capacitive systems. These advancements have enhanced deionisation capacity and provided molecular selectivity towards electrodes. Faradaic platforms have been engineered for aqueous purification, utilising the electrochemical reduction of specific contaminants, electrochemical ion exchange switching, and the discriminative elimination of ions, uncharged compounds, and biomolecules [51].

Emerging electrochemical methods include electrodeionization (EDI), shock electrodialysis, capacitive deionization, battery deionization, and electrosorption utilizing faradic compounds. These innovations differ from other methods as they remove contaminants depending on how they react to electrochemical processes at electrodes or electric fields in solutions. Electrochemical apparatus uses applied electrical currents to induce bulk electrolyte separations, trap impurities in EDLs, or intercalate them into solid electrodes. These systems are scalable without high pressures or temperatures because their primary energy source, voltage, has been applied. Ohmic resistance, resistance to the Faradaic reaction at electrode–electrolyte contacts, and concentration polarization contribute to energy dissipation in electrochemical systems [52]. Instead of scaling with the amount of solution treated, these losses vary according to how many ions are removed, resulting in a higher energy efficiency than physical methods when treating brackish water and diluted feeds [52].

While electrodialysis (ED) has undergone comprehensive research and widespread application in water desalination, innovative electrochemical systems with distinctive functionalities and operational principles have emerged in recent years. These include shock electrodialysis (shock ED), electrodeionization (EDI), capacitive deionization (CDI), and Faradaic electrosorption. Newman and Thomas-Alyea (2012) provided an overview of the development of these technologies. This laid the groundwork for the burgeoning domain of electrochemical systems dedicated to ion separation and water purification [52]. Additionally, microfluidic technologies driven by electrical energy were briefly discussed. However, their suitability for large-scale applications is limited compared to electrochemical systems, which offer clear pathways for scale-up through components such as electrodes, porous separators, and ion exchange membranes, which can be manufactured in substantial areas.

In an earlier study, Alkhadra et al. (2019) [40] explored the application of shock electrodialysis to continuously remove radionuclides from contaminated water [42]. Traditional methods for radionuclide removal, such as precipitation and adsorption, often require high removal efficiency and have many operational limitations. Alkhadra et al. (2019) [40] proposed that shock electrodialysis can effectively address these challenges. Shock electrodialysis is an emerging electrochemical method that uses electric fields to induce ion transport and separation in water [40]. This technique leverages the principle of ion concentration polarization to achieve the efficient and selective removal of radionuclides. In the study, the researchers designed and fabricated a shock electrodialysis cell consisting of an ion exchange membrane and a porous separator.

### 4.4. Bioremediation

Uranium reduction by microbes in sediments has been a vital research topic, as sediments play a crucial role in uranium’s fate and conveyance process in groundwater systems. Sediments act as both a source and a sink for uranium, providing a complex matrix for biogeochemical interactions and microbial processes. Understanding the biogeochemistry of uranium and the microbial communities involved in its reduction within different sediment types is vital for developing effective in situ groundwater remediation strategies.

Chung et al. (2014) [53] explored the role of microorganisms in the phytoremediation of ecosystems contaminated with uranium. The researchers investigated the presence of uranium-resistant bacterial populations in different environmental samples and analyzed their phylogenetic characteristics and metal resistance profiles. Notably, the sampling site with the highest uranium concentration had a significantly larger number of cultivable uranium-resistant bacteria, indicating the presence of an ecologically stable population of uranium-resistant organisms exhibiting adaptation to the challenging environment. By conducting cladistic analysis, the researchers identified distinct resistance profiles among different species of the Rhodanobacter genus. Notably, Rhodanobacter thiooxidans strains displayed resistance to uranium hexavalent and antimony pentavalent, while strains A2-61 and A2-60, also within the same group, exhibited resistance to zinc (II) and copper (II). Additionally, strain A2-442, classified as R. umsongensis, demonstrated resistance to uranium hexavalent and antimony pentavalent. It is worth mentioning that the type strains of these breeds did not exhibit any reported resistance to heavy metals. Rhodanobacter strains have undergone isolation from various uranium-polluted ecosystems; however, the factors contributing to their successful adaptation in such settings remain unclear. Oak Ridge Integrated Field Research Challenge strains resisted low-pH conditions. They possessed denitrification capabilities, which could hinder the in situ reoxidation of uranium tetravalent by reducing nitrate content. Furthermore, strain A2-61 within this genus demonstrated the ability to resist and aerobically reduce uranium hexavalent. Therefore, the prevalence of Rhodanobacter strains as some of the primary quarantines at the sampling station with an elevated uranium concentration suggests their significant involvement in shaping the fate and behavior of uranium in these environments.

Newsome et al. (2014a) [54] seemed to concur with Chung et al. (2014) [53], finding that the indigenous microbial communities in most sediments are capable of reducing uranium hexavalent to uranium tetravalent and effectively removing it from a solute undergoing redox reactions with an introduced electron donor [53,54]. The microbial reduction of uranium generally occurs through enzymatic action, where microorganisms utilize soluble U(VI) as a reducer and an electron acceptor to sparingly soluble U(IV). This transformation is favorable, as U(IV) precipitates in the sediment, immobilizing uranium in a less mobile and less toxic form. Microbial communities play a vital role in this process, reducing equivalents to convert U(VI) into U(IV). The availability of apt electron donors, such as organic matter or specific chemical species, influences the rate and efficiency of microbial uranium reduction. “Apt” electron donors, in this context, refer to electrons that can be readily utilized by microbes for the enzymatic reduction of U(VI) to U(IV), thereby facilitating efficient uranium immobilization. Additionally, the abundance and diversity of microorganisms capable of uranium reduction vary depending on environmental factors, including pH, redox potential, and other electron acceptors like nitrate or sulfate. Understanding the microbial ecology of uranium-contaminated ecosystems is essential for designing effective bioremediation strategies. By characterizing the microbial communities involved in uranium reduction and identifying the key players responsible for this process, scientists can develop targeted approaches to enhance microbial activity and uranium immobilization. Moreover, molecular techniques, such as DNA sequencing and gene expression analysis, allow researchers to gain insights into the functional potential of microbial communities and their response to changes in environmental conditions. To achieve the successful bioremediation of areas with uranium contamination, it is crucial to consider the complex interactions between microorganisms, the sediment matrix, and the contaminants. Integrating biogeochemical knowledge with engineering strategies can lead to the development of site-specific and cost-effective bioremediation plans that harness the natural capabilities of microbial communities to mitigate uranium contamination. In summary, the study of the microbial reduction of uranium in sediments and the interaction between microorganisms and uranium in contaminated ecosystems are vital for understanding the fate and transport of uranium in groundwater systems.

Recent advancements in electrochemical methods have led to the development of techniques such as shock electrodialysis and electrokinetics for efficient water purification and ion separation. These innovative methods offer advantages in selectivity and energy efficiency compared to conventional physical methods. As research in these fields progresses, further advancements in environmental remediation and water treatment technologies are anticipated. The study by Newsome et al. (2014b) [54] investigated the ability of different sediments from the Sellafield nuclear site in northwest England to establish an anaerobic environment and eliminate uranium hexavalent from a solution, demonstrating the occurrence of a series of anaerobic reduction–oxidation (redox) reactions encompassing nitrate, Fe (III), and sulfate reduction during the incubation period in most sediments [54]. Molecular analysis revealed the presence of microbial populations capable of utilizing an extensive spectrum of species capable of donating and accepting electrons, suggesting their involvement in diverse biogeochemical processes [55].

Although specific uranium hexavalent-reducing bacterial genera, such as Geobacter and Shewanella, were identified, their contribution to uranium hexavalent reduction remains unclear. The study also discussed the influence of sediment composition on uranium hexavalent reduction. While calcium, which can form stable Ca-uranyl-carbonate complexes, did not inhibit microbial uranium hexavalent reduction in the sediments tested, sorption effects were observed in clay sediments. The clay sediments exhibited uranium hexavalent reduction through sorption and microbial reduction, indicating their potential to provide natural uranium hexavalent attenuation. However, sediments with low bioavailable Fe (III) showed limited uranium hexavalent reduction, suggesting the importance of Fe (III)-reducing microbial communities. These findings indicate that stimulated microbial uranium hexavalent reduction could be a viable technique for the in situ remediation of groundwater exhibiting elevated levels of uranium at the Sellafield site and other UK nuclear sites with different lithologies.

Further research developments, including performing column chromatography at a pilot scale before deployment in the field, will be necessary if uranium remediation in groundwater becomes a priority. However, Newsome et al. (2014b) [54] presented a contrasting perspective [54]. The scientists agreed that extensive investigations have been carried out on uranium biogeochemistry and bioremediation, and the application of bioreduction, in particular, has emerged as a viable technique. Novel molecular methodologies are being devised to track advancements and optimize their implementation in the field. Furthermore, field tests have demonstrated the persistent removal of uranium hexavalent from groundwater. They asserted that there are still uncertainties regarding the durability of decreased uranium tetravalent. Although biomineralization has been shown to produce uranyl phosphates that are weakly soluble using pure bacterial cultures, sorption effects have dominated results with soils from the Oak Ridge site. Strontium and technetium are two other priority radionuclides that may be treated through bioreduction or biomineralization. One area where studies should be pursued is assessing the dominant microorganisms that transfer electron systems in natural ecosystems and during biostimulation experiments. The specific transfer of electron mechanism(s) to uranium hexavalent in circumneutral aquifer sediments is being determined. More research is necessary to determine whether bacteria can enzymatically reduce solid-phase uranium hexavalent and Gram-positive bacteria’s role in reducing uranium hexavalent at an alkaline pH. The duration of reduced uranium tetravalent is another topic for future studies. Field biostimulation trials could include reoxidation experiments to evaluate the above area. However, it is essential to comprehend the mechanisms leading to monomeric uranium tetravalent or uraninite precipitation. Still, it is unclear whether monomeric uranium tetravalent is unique to that site or has broader significance, because the phenomenon is absent from the geological record [55]. Laboratory experiments have not provided conclusive proof of an aging mechanism. This might be investigated in field research conducted in different locations and nations. One area that needs more research is the uranium contamination of groundwater at mining or milling sites.

### 4.5. Permeable Reactive Barrier

**Figure 7:**
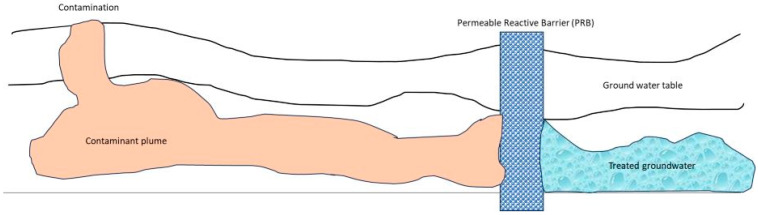
Description of the technique of permeable reactive barrier (PRB).

Groundwater contamination poses a significant environmental challenge, requiring effective and sustainable remediation strategies. Traditional approaches like pump-and-treat systems have cost and long-term energy-intensive operation limitations [55,56]. As a result, the development of innovative remediation technologies has become crucial. One emerging technology that has been deeply studied is the permeable reactive barrier (PRB), as schematically described in Figure 7.

PRBs are passive in situ remediation systems constructed underground, utilizing reactive materials to intercept and treat groundwater contaminant plumes. This technology offers several advantages, including the ability to treat contaminants and reduce their concentrations below detection limits. Naidu and Birke (2015) [55] discussed the challenges associated with groundwater remediation and the limitations of conventional pump-and-treat systems. Naidu and Birke (2015) [55] emphasized the need for tailored approaches and multiple remediation technologies to address the complexity of contaminated sites. In addition, they highlighted the emergence of PRBs as an effective and sustainable in situ remediation technology [56]. However, choosing the most suitable mitigation strategy depends on site conditions, contaminant types, and impacts. Often, a combination of technologies, known as “treatment trains”, is necessary for effective site remediation.

The past two decades have observed significant advancements and progress in site treatment technologies, including groundwater circulation wells and PRBs, which have gained popularity in Europe and the United States. Discussing the design of PRBs, Chamberlain et al. (2011) [56] outlined that the process involves the creation of a vertical permeable utilizing a reactive substance in the vadose zone beneath the surface [56]. They are designed to intercept the groundwater contaminant plume and treat it as it flows through the barrier. The permeability of PRBs allows for the passage of groundwater without significantly altering hydrogeology while trapping or degrading contaminants [56]. Due to minimal above-ground disruption, PRBs are particularly suitable for urban environments and built-up areas. Their design depends on site-specific conditions, where the following two possible configurations may be implemented: continuous barriers and “funnel-and-gate” systems [56]. The selection of reactive material depends on the type of contaminants and groundwater composition. Feasibility studies and high-resolution site characterization play crucial roles in PRB design. Naidu and Birk (2015) [55] concluded that PRBs have effectively treated various contaminant types and are considered to be a sustainable remediation technology [55]. PRBs can reduce contaminant concentrations to below detection limits [56]. However, PRBs do not apply to all site conditions, and a thorough assessment is required to determine their suitability.

### 4.6. Aeration Technique

The presence of radon gas in water sources poses health risks, making its removal a crucial concern. Among the available techniques, aeration is emerging as an effective method for eliminating radon from water. Still, it also releases radon gas into the surrounding air, thus posing health risks if this occurs in closed spaces. Aeration involves exposing water to the air or increasing its contact with the atmosphere, allowing radon gas to disperse in the air. This process takes advantage of radon’s tendency to escape from water when readily exposed to air or agitated. Aeration methods have effectively eliminated radon from water sources, including diffused bubble aeration, packed tower aerators, spray nozzle aeration, and gas–degas technology (GDT).

Earlier, Montaña et al. (2013) [43] discussed some of the techniques used in aeration methods, such as diffused bubble aeration, packed tower aeration, Venturi aerator aeration, and spray nozzles for radon removal, analyzing their efficiencies in different waterworks across Finland, Sweden, and Germany. The radon concentrations in the raw water samples varied. However, aeration consistently removed radon and carbon dioxide. The study concluded that the most effective radon removal rates (98–100%) can be achieved by optimizing aeration processes using gas–degas technology (GDT), packed tower aerators, and spray nozzles [43].

Similarly, Jastaniah et al. (2014) [57] explored methods for removing radon from an aqueous solution [58]. These authors found that the diffused bubble aeration method can effectively remove more than 98% of radon emanating from artificially enhanced aqueous solutions. Additionally, it was discovered that heat is effective in removing radon, with a temperature of 39 °C and a duration of 5 h, resulting in a removal rate of approximately 76%. Jastaniah et al. (2014) [57] also investigated the optimum airflow rate for removing radon-222 from different water volumes [57]. Jastaniah et al. (2014) [57] determined that, for a 50 mL water volume, an airflow rate of 242 mL/min for an aeration time of 1.5 min reduced the relative remainder of ^222^Rn in water to about 6% [57].

Similarly, for a 100 mL water volume, an airflow rate of 345 mL/min achieved a 14% reduction in radon remainder within the same aeration time. The optimal aeration time for radon removal was 3 min for volumes of 50 mL and 100 mL and 10 min for volumes of 200 mL and 300 mL [57]. Furthermore, Jastaniah et al. (2014) [57] observed that the natural loss of radon from water rich in radon increased with a larger surface area [57].

### 4.7. Activated Carbon

Activated carbon (AC)-based adsorption techniques have great potential for treating radionuclide-contaminated water due to their simple design, high efficiency, wide pH range, quickness, low cost, reusing procedure, and environmental friendliness [58].

Alabdula’aly et al. (2011) [59] described the performances of three types of carbon products, specifically, Filtrasorb 300 (F-300), Filtrasorb 400 (F-400), and Hydro Darco 4000 (HD-4000), in removing radon from water containing an average activity level of 96 Bq dm^−3^. All three types of carbon were sieved, and the fraction between 1.0 and 1.4 mm was selected arbitrarily [59]. This was conducted to keep all granular-activated carbon (GAC) grain sizes in the same range and compare their performance uniformly [59]. The results showed a range of radon removal between 92% and 99.4% [59]. Among the wide range of uses of activated carbon, it is a reliable choice for removing radionuclides from water. However, some limitations can be addressed, like the accumulation of radionuclides and the treatment method of spent GAC. Almost all water treatment plants worldwide have introduced activated carbon into their treatment procedure due to its effectiveness in decontaminating water.

### 4.8. Comparative Costs of the Different Techniques

When considering water treatment processes for radionuclide removal, the economic factor of cost becomes a crucial aspect. In general, activated carbon adsorption aids in eliminating radon, radium, and uranium, and reverse osmosis (RO) is employed to separate and remove radium and uranium. Among these processes, RO was found to have the highest costs. Munter (2013) [60] provided, in his study, a cost analysis, revealing that the aeration process for ^222^Rn removal had a cost range from EUR 0.009 to 0.018 per cubic meter (euros/m^3^) [60]. On the other hand, the cost range for activated carbon adsorption, including annual regeneration, was from 0.007 to 0.008 EUR/m^3^ [60]. In comparison, the cost range for reverse osmosis was higher, ranging from 0.092 to 0.11 EUR/m^3^ [60]. Factors like energy consumption, retention time, and regeneration method affect the total cost of each treatment. In addition to data from the Milano aqueduct, further cost information from the United States was provided [60]. The expenses associated with lime softening, ion exchange, and MnO2-based methods ranged from 0.07 to 0.19 EUR/m^3^ [60]. Using radium-selective adsorbents is a relatively cheaper option, with costs ranging from 0.04 to 0.09 EUR/m^3^ [60]. In light of preliminary estimates, the optimized iron–manganese filtration process, patented by Water Technology Partners Ltd., Estonia, has emerged as one of the most cost-effective options for radium removal [60]. This process is estimated to have a preliminary total cost of approximately 0.02 EUR/m^3^, indicating that it can have an economic advantage in radionuclide removal compared to other methods [60].

## 5. Conclusions

This literature review has comprehensively described state-of-the-art methods for removing naturally occurring radioactive materials from water. Reverse osmosis, electrodialysis, iron manganese filtration, and membrane filtration show varied efficiencies in removing radionuclides. However, each technique has a dedicated use case depending on the nature of the radionuclide to be removed and whether the method suits the project’s design, cost, and objectives. 

The main factor in selecting a removal method depends on the radioactive material’s physical and chemical features [61]. Furthermore, cost-related studies showed differences in price related to different removal techniques; for instance, reverse osmosis shows the highest costs in the removal process, while the iron–manganese filtration process has proven to be the cheapest among the other methods. Additionally, the potential for combining multiple techniques into a treatment train could provide more efficient and cost-effective solutions for removing radionuclides from water. Further studies, including laboratory work and pilot-scale tests, are needed to determine the most suitable, cost-effective, and environmentally friendly method. Sustainable and green materials can also be studied further for their capability in removing radionuclides from water.

## Figures and Tables

**Table 1:** Physicochemical characteristics of radon, radium, thorium, and uranium.

Material	Reactivity	Solubility	Mobility	References
**Radon**	Chemically inert noble gas. However, its short-lived decay products, like polonium, are chemically reactive and easily attached to aerosols and particulate matter in the air.	Not soluble in water as a gas. Its decay products, mainly polonium-210, may be soluble, deposited on surfaces, or dissolved in water.	Easily dispersed in the gaseous state and can penetrate through different materials such as soil and construction. Its mobility is influenced mainly by diffusion and pressure gradients in the soil or building materials.	[8,9,10,11]
**Radium**	A reactive chemical element within the alkaline earth group that can combine with other elements to form a chemical compound [5]. Its reactivity varies with the chemical form and or condition in which the chemical is present.	Generally insoluble in water at neutral pH levels. It dissolves well in water at ordinary temperatures, except in cases where the pH is low or the salinity is high [6].	Exists as a free phase in the environment, often in groundwater. Radium mobility can be affected by factors such as the acidity level, temperature, and other chemicals in the water or soil.	[12,13]
**Thorium**	It is capable of actively combining with other elements to form a compound. It also has variable oxidation states and reacts with oxygen, acids, and other chemical reagents.	Its solubility may fluctuate based on the type of form, including in the diet and other situations that characterize one’s environment. Like most elements, thorium can form both insoluble and soluble compounds with water.	Very mobile in some settings, particularly in an acidic environment. Its mobility in groundwater and surface water can contaminate soil and water sources.	[14,15]
**Uranium**	React with other elements and can exist in different oxidation states. It is most relevant in the areas of nuclear energy and pollution.	Solubility of compounds in water can be variable depending on the chemical form and the conditions of the environment. Depending on the pH and redox conditions, uranium can dissolve in water to form soluble and insoluble complexes.	Can be mobile in the environment, especially in groundwater and surface water. Its mobility is influenced by factors such as pH, temperature, and the presence of another chemical. Uranium contamination can also cause the element to be transported over vast distances and affect the environment.	[16,17,18]

**Table 2:** Uranium-238 decay series [6].

Element	Half-Life	Emitted Radiation
Uranium-238	4.468 billion years	Alpha
Thorium-234	24.1 days	Beta
Protactinium-234	1.17 min	Beta
Uranium-234	245,500 years	Alpha
Thorium-230	75,380 years	Alpha
Radium-226	1600 years	Alpha
Radon-222	3.823 days	Alpha
Polonium-218	3.05 min	Alpha
Lead-214	26.8 min	Beta
Bismuth-214	19.9 min	Beta
Polonium-214	164 microseconds	Alpha
Lead-210	22.2 years	Beta
Bismuth-210	5102 days	Beta
Polonium-210	138 days	Alpha
Lead-206 (stable)	N/A	N/A

**Table 3:** Thorium-232 decay series [6].

Element	Half-Life	Emitted Radiation
Thorium-232	14.05 billion years	Alpha
Radium-228	5.75 years	Beta
Actinium-228	6.13 h	Beta
Thorium-228	1.913 years	Alpha
Radium-224	3.62 days	Alpha
Radon-222	55.62 s	Alpha
Polonium-216	0.146 s	Alpha
Lead-212	10.463 h	Beta
Bismuth-212	60.55 min	Alpha, Beta
Polonium-212	0.298 microseconds	Alpha
Thallium-208	3.053 min	Beta
Lead-208 (stable)	N/A	N/A

**Table 4:** Uranium-235 decay series [6].

Element	Half-Life	Emitted Radiation
Uranium-235	0.71 billion years	Alpha
Thorium-231	25.6 h	Beta
Protactinium-231	33,000 years	Alpha
Actinium-227	22 years	Alpha, Beta
Thorium-227	18.2 days	Alpha
Francium-223	22 min	Beta
Radium-223	11.7 days	Alpha
Rodon-219	3.9 s	Alpha
Polonium-215	1.8 microseconds	Alpha, Beta
Lead-211	36 min	Beta
Astatine-215	1.8 milliseconds	Alpha
Bismuth-211	2.16 min	Alpha, Beta
Thallium-207	4.8 min	Beta
Polonium-211	0.52 s	Beta
Lead-207(stable)	-	-

**Table 5:** Water quality guidelines regarding radionuclides in the US, Europe, and Jordan [29,30,31,32].

United States	
**Radionuclides**	**Maximum Contaminant Level ^1^**
Combined Radium-226 and Radium-228	185 Bq/m^3^
Gross Alpha (excluding 222Rn and uranium)	555 Bq/m^3^
Uranium	0.372 Bq/m^3^
**Europe**	
**Parameter**	**Parametric Value ^2^**
Tritium	100,000 Bq/m^3^
Rn-222	100,000 Bq/m^3^
Indicative Dose	0.1 mSv
**Nuclide**	**Derived Concentration ^3^**
Radium-226	500 Bq/m^3^
Radium-228	200 Bq/m^3^
Lead-210	200 Bq/m^3^
Uranium-234	2800 Bq/m^3^
Uranium-238	3000 Bq/m^3^
Polonium-210	100 Bq/m^3^
**Jordan**	
**Radioactive Material**	**Standard level ^4^**
Alpha Particles (excluding Rn-222) *	500 Bq/m^3^
Beta Particles (excluding Carbon-14 and Tritium) **	1000 Bq/m^3^

^1^ The highest level of a contaminant that is allowed in drinking water. MCLs are set as close to MCLGs (maximum contaminant level goals) as feasible using the best available treatment technology and taking cost into consideration. MCLs are enforceable standards. ^2^ Refers to the value of radioactive substances in water intended for human consumption. ^3^ This refers to a theoretical value [derived mathematically] for the maximum allowable concentration of a specific radionuclide in drinking water. ^4^ Standard levels are reference values rather than maximum limits. If beta and alpha radionuclides exceed the standard level, relevant agencies should investigate the source and form of radionuclides. * If alpha and beta radionuclides exceeded the reference level of radioactive properties, an investigation of the type and source of the radionuclide should be carried out, as well as an assessment of health effects, to measure the level of exposure to radioactive materials so that it does not exceed 0.1 millisevert/year for Beta for each element [32]. ** Excluded items are not computed under the reference level [32].

**Table 6:** Summary of different water treatment techniques for removing radium, uranium and radon from water [41].

Treatment Technique	Removal Techniques	Removal Efficiency
Radium	Coagulation/filtration	65–95%
	Membrane processes	90–99%
	Lime softening	80–95%
Uranium	Coagulation/filtration	80–95%
	Lime softening	85–99%
	Membrane processes	90–99%
Radon	Aeration system	70–99%
	Packed tower	90–99%
	Granular activated carbon	80–99%

**Table 7:** Typical reverse osmosis treatment stages.

Stage	Component	Function
1	Pre-filter, spun-bounded polypropylene	Removes suspended impurities like particles of rust and dust
2	Pre-carbon filter, silver-impregnated granular-activated carbon	Removes color, odor, and free chlorine and absorbs organics and pesticides
3	Sediment filter, spun PP cartridge	Acts as a final filter to remove smaller contaminants and remaining particles. Reduces fine turbidity
4	Reverse osmosis membrane thin-film composite (TFC) ~0.0001 micron	Removes TDS, hardness, fluoride, pesticides, heavy metals like lead, mercury, cadmium, uranium, and arsenic, etc., and micro-organisms like bacteria, viruses, and protozoan cysts
5	Post-carbon filter, silver-impregnated fine granular-activated carbon	Imparts bacteriostatic property and helps in reviving the original taste of water

**Table 8:** Comparing GO and reverse osmosis.

Feature	Graphene Oxide (GO) Membranes	Membrane Technology (RO)
Mechanism	Size-sieving, tunable ion separation	Pressure-driven separation, size, and charge exclusion
Selectivity	High, tunable	Lower
Water Flux	High in some cases	Generally lower than GO membranes
Energy Consumption	Potentially lower	Can be high
Fouling	Prone to fouling	Prone to fouling
Applications	Separation of radionuclides in complex mixtures and highly acidic or saline conditions	Treatment of radionuclide-containing wastewater, water purification

## Data Availability

Data are contained within the article.
